# Ubiquitin-Like Post-Translational Modifications (Ubl-PTMs): Small Peptides with Huge Impact in Liver Fibrosis

**DOI:** 10.3390/cells8121575

**Published:** 2019-12-04

**Authors:** Sofia Lachiondo-Ortega, Maria Mercado-Gómez, Marina Serrano-Maciá, Fernando Lopitz-Otsoa, Tanya B Salas-Villalobos, Marta Varela-Rey, Teresa C. Delgado, María Luz Martínez-Chantar

**Affiliations:** 1 Liver Disease Lab, CIC bioGUNE, Centro de Investigación Biomédica en Red de Enfermedades Hepáticas y Digestivas (CIBERehd), 48160 Derio, Spain; slachiondo@cicbiogune.es (S.L.-O.); mmercado@cicbiogune.es (M.M.-G.); mserrano@cicbiogune.es (M.S.-M.); mvarela.ciberehd@cicbiogune.es (M.V.-R.); mlmartinez@cicbiogune.es (M.L.M.-C.); 2 Liver Metabolism Lab, CIC bioGUNE, 48160 Derio, Spain; flopitz@cicbiogune.es; 3 Department of Biochemistry and Molecular Medicine, School of Medicine, Autonomous University of Nuevo León, Monterrey, Nuevo León 66450, Mexico; bernardette.salas@gmail.com

**Keywords:** Ubiquitination, NEDDylation, SUMOylation, HCC, chronic liver disease, NAFLD, NASH

## Abstract

Liver fibrosis is characterized by the excessive deposition of extracellular matrix proteins including collagen that occurs in most types of chronic liver disease. Even though our knowledge of the cellular and molecular mechanisms of liver fibrosis has deeply improved in the last years, therapeutic approaches for liver fibrosis remain limited. Profiling and characterization of the post-translational modifications (PTMs) of proteins, and more specifically NEDDylation and SUMOylation ubiquitin-like (Ubls) modifications, can provide a better understanding of the liver fibrosis pathology as well as novel and more effective therapeutic approaches. On this basis, in the last years, several studies have described how changes in the intermediates of the Ubl cascades are altered during liver fibrosis and how specific targeting of particular enzymes mediating these ubiquitin-like modifications can improve liver fibrosis, mainly in in vitro models of hepatic stellate cells, the main fibrogenic cell type, and in pre-clinical mouse models of liver fibrosis. The development of novel inhibitors of the Ubl modifications as well as novel strategies to assess the modified proteome can provide new insights into the overall role of Ubl modifications in liver fibrosis.

## 1. Chronic Liver Disease (CLD)

Liver injury induces inflammation, necrosis of hepatocytes, angiogenesis, the wound-healing response and the accumulation of extracellular matrix (ECM) proteins; followed by a process of hepatocyte regeneration to replace dead hepatocytes and restore the physiological liver mass [1,2]. Liver fibrosis typically reverts after elimination of the causative injury. However, if the damage persists and a chronic response is established, liver fibrosis can progress to cirrhosis, which is characterized by the distortion of the hepatic parenchyma and vascular structures that can eventually lead to hepatic loss of function and potential loss of reversibility [3]. At this stage, if the injury is not withdrawn, patients are at risk of end-stage liver disease and complications such as portal hypertension, hepatocellular carcinoma (HCC), and liver failure [3,4].

### 1.1. Etiology and Pathophysiology of Chronic Liver Disease (CLD)

Chronic liver disease (CLD) affects 800 million people worldwide and accounts for approximately 2 million deaths worldwide annually, representing a global major public health issue [4,5,6]. Alcohol abuse and associated alcoholic liver disease (ALD), viral hepatitis, and non-alcoholic fatty liver disease (NAFLD) are the most common causes of CLD. However, inherited disorders (such as alpha antitrypsin deficiency, hemochromatosis, and cystic fibrosis), drugs, cholestatic disease (such as primary biliary cholangitis (PBC), and primary sclerosing cholangitis (PSC)), and immune disorders also contribute to this common pathology [4].

Alcoholic liver disease (ALD) and NAFLD share a similar pathological progression, ranging from simple steatosis to alcoholic steatohepatitis (ASH) or non-alcoholic steatohepatitis (NASH). fibrosis, cirrhosis, and HCC [7]. Whereas excessive alcohol consumption is the main cause of ALD, the pathogenesis of NAFLD is related with obesity, insulin resistance and/or the metabolic syndrome, gut microbiota dysbiosis, environmental or nutritional factors, and genetic and epigenetic factors (reviewed by [8]). On the other hand, cholestatic disease [such as primary biliary cholangitis (PBC) and primary sclerosing cholangitis (PSC)] are associated with chronic damage to the cholangiocytes of the biliary tree, leading to reductions in the bile flow, persistent injury to the biliary epithelium and hepatocytes, inflammation, fibrogenesis and potentially carcinogenesis [4,9].

### 1.2. Liver Fibrosis and Cell Types

Liver damage leads to death of hepatocytes and cholangiocytes, which induces the release of pro-inflammatory mediators and stimulates phagocytosis of dead cell bodies by liver macrophages, mainly Kupffer cells and bone marrow-derived recruited monocytes [10]. Macrophages in the liver can also produce pro-inflammatory factors, such as reactive oxygen species (ROS), CC-chemokine ligand 2 (CCL2), tumor necrosis factor (TNF), interleukin-6 (IL-6), and 1β (IL-1β), thus triggering the wound-healing response, and stimulating the production of extracellular matrix components by myofibroblasts [11].

In ALD, the hepatocyte injury is mainly related to the oxidative metabolism of ethanol, whereas in NAFLD it depends on the lipotoxicity that induces cell death and lipo-apoptosis. When liver fibrosis is developed in an onset of ALD or NAFLD, the excessive deposition of ECM proteins is principally observed around the sinusoids (peri-sinusoidal fibrosis) and around groups of hepatocytes (peri-cellular fibrosis), and is mainly due to hepatic stellate cells (HSC) [4,12]. When fibrosis is developed in an onset of cholestasis, in addition to chronic damage to cholangiocytes, bile acids elicit hepatocyte injury and death [4,13,14]. In chronic diseases of the biliary tract, the excessive deposition of ECM proteins is principally observed around the injured bile ducts (biliary fibrosis pattern) and is mainly characterized by the proliferation of reactive ductular cells and myofibroblasts originated from portal fibroblast and HSC [4,15,16]. However, the contribution of portal fibroblasts to the development of fibrosis after cholestatic damage, compared to that of the HSC, is controversial [17].

As mentioned before, despite other minor cell sources (reviewed by [4]), HSC are the main sources of myofibroblasts in response to toxic liver injury [18,19]. In a healthy liver, HSC are in a quiescent state in which they accumulate retinoids. In response to toxic liver injury, HSC suffer a transdifferentiation process from a quiescent into an activated phenotype known as myofibroblasts [20]. These activated HSCs have a higher degree of proliferation and migration, hence repopulating the damaged liver, acquiring contractility by expressing alpha smooth muscle actin (α-SMA), expressing pro-inflammatory [(monocyte chemoattractant protein-1 (MCP-1), platelet-derived growth factor (PDGF), mouse stem cell factor (mSCF), CCL2, and CCL21, as well as IL-1β) and pro-fibrogenic markers (TGF-β)], and as well as increasing the synthesis of ECM proteins [collagen I (COL1A1) and III (COL1A3), fibronectin and tissue inhibitor of metalloproteinase (TIMP)], and of pro-angiogenic mediators [like vascular endothelial growth factor A (VEGFA), angiopoietin-1 or -2, and the homodimer (PDGF-BB)] [21,22]. One of the principal factors involved in HSC-induced proliferation is PDGF, which is upregulated in the fibrotic liver, whereas transforming growth factor (TGF-β) is the main profibrogenic factor and contributes positively to the transdifferentiation process of HSCs into myofibroblasts. Briefly, TGF-β binds and activates TGF-β receptors (TβR), of which there are three different forms (TβRI, TβRII, and TβRIII). Smads are the effector proteins of the TGF superfamily ligands. There are 8 Smad proteins which include: receptor-regulated R-Smads (Smads 1, 2, 3, 5, and 8), common-mediator Co-Smads (Smad4), and inhibitory I-Smads (Smads 6, 7). When TβRI is activated, Smads are recruited to the receptor and phosphorylated, resulting in their activation and increased affinity for Smad4. Then the Smad2/3/4 heteromeric complex translocates to the nucleus, where it has an immediate effect on the gene expression of several hundred of genes. TGF-β signaling is terminated when the activated Smads are either dephosphorylated or degraded [23,24].

### 1.3. Overview of the Current Treatment Options for Chronic Liver Disease and Liver Fibrosis

Current therapeutic interventions targeting CLD are etiology-dependent. Whereas in the last years, a quantum leap has been made in the therapy of hepatitis-induced CLD thanks to the development of novel and effective anti-viral drugs [25,26], therapeutic interventions in the case of ALD remain abstinence, treating the alcohol withdrawal syndrome, nutritional support, glucocorticosteroids or Pentoxifylline, anti-TNF therapy, antioxidants, or liver transplantation. On the other hand, treatment options for NAFLD and NASH are mainly directed toward lifestyle changes and weight loss, in combination with drugs such as insulin sensitizers, lipid lowering agents, hepatoprotective agents, antioxidants, incretin analogues, and anti-inflammatory agents (reviewed by [7]). First-line treatment for PBC involves the ursodeoxycholic acid (UDCA), which is able to prevent the progression of the disease in approximately two thirds of the patients [27,28,29]. For those PBC patients with insufficient response or intolerance to UDCA, second-line therapy is obeticholic acid [30,31]. There is currently no effective pharmacological therapy for PSC being liver transplantation the most definitive treatment [32].

Importantly, current clinical guidelines reinforce that, independently of the etiology of CLD, liver fibrosis should be the pharmaceutical target stage, once liver fibrosis reversibility is a reality. Taking into consideration that liver fibrosis is a multi-step disease characterized by pan-cellular and pan-pathway mechanisms, post-translational modifications (PTMs) of proteins can provide a better understanding of the liver fibrosis pathology as well as novel and more effective therapeutic approaches.

## 2. Post-Translational Modifications (PTMs) by Ubiquitin-Like (Ubl) Proteins

Post-translational modifications (PTMs) of proteins play a relevant role in the functional diversity of the proteome. In most eukaryotes, PTMs refer to the covalent and reversible addition of small chemical entities into target proteins following protein biosynthesis in order to exert a dynamic control over protein function in diverse cell biological contexts. The recent advances in the fields of systems biology and proteomics, have pushed forward the interest in deciphering protein modifications and their impact on the cellular microenvironment and disease pathophysiology. The most common PTMs include phosphorylation, acetylation, glycosylation, ubiquitination, acetylation, and hydroxylation, among others.

Ubiquitination is implicated in the pathogenesis of certain human diseases, including liver fibrosis. Ubiquitin has shown to be a marker of non-alcoholic liver fibrosis and it is frequently detected at the border or within the fibrous matrix [33,34]. Under these circumstances, overall changes in the ubiquitinated proteome may reflect either modifications in the ubiquitination cascade or in the proteasomal activity. For example, Cai et al. detected, in a rat model of liver fibrosis, a reduced SMAD specific E3 ubiquitin protein ligase 2 (Smurf-2) mRNA expression, which is a HECT domain E3 Ub ligase that ubiquitinates nuclear Smads and targets them for proteasomal degradation, resulting in an increased Smad2 expression [35]. Gp78, an endoplasmic reticulum (ER)-associated E3 Ub ligase, is also a key player in the ER-associated degradation (ERAD) and responsible for ubiquitination of lipid metabolism mediators, among others. Loss of Gp78 in aged mice caused NASH with fibrosis as a result of spontaneous and random ER stress [36]. On the other hand, Wilson and colleagues have shown that the Ub C-terminal hydrolase L1 (UCHL1) is an absent DUB in quiescent HSCs but its expression is increased and positively correlates with HSC transdifferentiation, in pre-clinical mouse models and in human livers from NASH and ALD patients. Pharmacological inhibition of UCHL1 in CCl_4_ and bile-duct ligated (BDL) mice or ablation of UCHL1 in vitro in cultured HSC cells reduces liver fibrogenesis [37]. Likewise, an increase in mRNA expression and immunoreactivity of synoviolin which is an E3 Ub ligase has been observed in myofibroblasts. Fibrotic human livers also showed co-localization of synoviolin and the main fibrotic marker, α-SMA [38]. This compelling evidence implicating ubiquitination in liver fibrosis led several authors to evaluate the impact of ubiquitin-like proteins (Ubls)-mediated PTMs in liver fibrosis, the topic of this Review.

Ubiquitin-like proteins (Ubls) are a family of small proteins involved in PTMs, whose name is derived from ubiquitin, the first discovered member of the family. Besides ubiquitin, the human genome encodes at least eight families of Ubls, that are considered type I Ubls: (SUMO) small ubiquitin-related modifier, NEDD8 (neural precursor cell expressed developmentally downregulated protein 8), ATG8 (autophagy-related protein 8), ATG12 (autophagy-related protein 8), URM1 (ubiquitin-related modifier 1), UFM1 (ubiquitin-fold modifier 1), FAT10 (human leukocyte antigen-F adjacent transcript 10 or ubiquitin D), and ISG15 (interferon-stimulated gene 15) [39]. Even though, sparse studies have shown alteration of the levels of some Ubls in liver fibrosis, namely ATG12 related to autophagy [40], Fat10 and UFM1 [41], and ISG15 specifically in hepatitis C [42], in this Review, we will specially focus on the relevance of NEDD8 and SUMO proteins in liver fibrosis, whose therapeutic role has been addressed in liver fibrosis. The main characteristics of these proteins in comparison to ubiquitin can be found in Table 1.

### 2.1. NEDDylation in Liver Fibrosis

NEDDylation is a reversible ubiquitin-like PTM, characterized by the covalent conjugation of NEDD8. The pathway of the NEDDylation process involves NEDD8 specific enzymes, such as E1 activating enzymes (NAE1 and UBA3); E2 conjugating enzymes (UBE2M/UBC12 and UBE2F); E3 ligase enzymes, which catalyze NEDD8 transference to the target protein (MDM2, RBX1, FBXO11, RNF7, CBL, DCUN1D1, and DECUN1D2); and deneddylase enzymes (SENP8/NEDP1, ATXN3, USP21, CPS5, UCHL1, and UCHL3) [47,48]. Noteworthy, NEDD8 is synthetized as a precursor and must be activated at the C-terminal Gly76 mainly by NEDP1 [49] in order to be integrated inside the NEDDylation cycle and conjugated to the lysine residue of target proteins [50]. The conjugation of NEDD8 can modify its target protein in different ways, such as inducing conformational changes, changing its subcellular localization, enzymatic activation, or inhibition, competing with other Ubls or inducing its stability [51,52].

The mechanisms that trigger the deregulation of NEDDylation are not well understood, but it has been reported that the levels of NEDD8 are increased under stress conditions in vitro [53]. In fact, alterations in the NEDDylated protein levels have been described in different pathological conditions, such as neurodegenerative disorders [54] and cancer [48,55,56]. Focusing on the liver context, patients with HCC and intrahepatic cholangiocarcinoma, as well as mouse models of HCC, showed a significant increase in the global NEDDylation proteome and NEDDylation intermediates [55,56,57,58,59]. In addition, under diverse stress conditions, the canonical pathway of NEDDylation via NAE1 changes, being NEDD8 conjugation predominantly mediated by the Ube1 E1 ubiquitin enzyme [53]. Likewise, in HCC, where NEDDylation levels are enriched, NEDP1 protein levels disappear promoting the inhibition of ATPase activity of HSP70 and, thus the apoptosis resistance of cancer cells. Hence, these result shows how the tight regulation of the NEDD8 cycle can modulate vital cellular functions like apoptosis [60].

Regarding liver fibrosis and NEDDylation, Zubiete-Franco et al. described for the first time an increase in the global NEDDylated proteome in patients with liver fibrosis as well as in mouse models of CCl_4_- and BDL-induced liver fibrosis [61]. Importantly, NAE1-specific inhibition in these mouse models showed a reduction in the liver damage associated with decreased apoptosis, inflammation, and fibrosis. These results were explained by the effect of NEDDylation inhibition in the different hepatic cell subtypes. The decrease in inflammation after NEDDylation inhibition can be explained in part by the incapacity of Cullin-1 and SCF^βTrCP^ (E3 Ligase) to ubiquitinate and degrade IKBα, promoting NF-kB stabilization in the cytoplasm [47,48]. Interestingly, in this work the authors describe how NEDD8 levels increase in activated HSCs, and consequently neddylation inhibition could directly block its activation. Indeed, after NAE1 inhibition, HSCs show an increase of cell death partly mediated by c-Jun accumulation, a target of cullin degradation. On the other hand, it has been described that Casitas B-lineage lymphoma (c-Cbl) acts as a NEDD8 Ligase promoting TGF-β signaling and stabilization of the type II receptor (TβRII) in blood cells [62]. In agreement with this line of evidence, other authors have shown very recently that the in vivo inhibition of the transcription factor SRSF3 NEDDylation, associated with its prevention of degradation, protects mice from fibrosis [63].

In conclusion, the NEDDylation inhibition is a key mechanism to down-regulating the inflammatory response, further reducing cell damage and subsequent liver fibrosis, in addition to specifically targeting HSC death.

### 2.2. SUMOylation in Liver Fibrosis

SUMOylation is another ubiquitin-like PTM that consists in the covalent addition of one or multiple SUMO subunits to Lys residues usually located on the SUMO consensus motifs of target proteins. SUMOylation occurs as a hierarchically organized process catalyzed by the E1 activating enzyme, the E2 conjugating enzyme, and an E3 SUMO ligase [64]. The extension of the SUMO chain is possible thanks to a specialized type of E3 ligase family of enzymes known as E4 SUMO elongases [65].

To date, five SUMO isoforms have been described in humans, being SUMO 1, 2, and 3 the most ubiquitous. SUMO modifiers are similar in size and structure to ubiquitin, but show little sequence homology compared to ubiquitin. SUMO 2 and 3 share approximately 97% identity, whereas SUMO 1 is only 50% identical in sequence. SUMO isoforms differ in several aspects, such as in the E3 ligase preference or the ability to form SUMO chains on the substrate proteins. Moreover, different functions and mechanisms of regulation within the cell would be expected since SUMO2/3 conjugation becomes more relevant under stress conditions [64]. The SUMO E1 activating enzyme is composed by the SAE1 and UBA2 heterodimer, while Ubc9 is the only E2 SUMO conjugating enzyme recognized. Conversely, a huge range of E3 SUMO ligases exist, which are grouped in the canonical PIAS family and non-canonical E3 ligases such as RanBP2 or Cbx4, thus conferring specificity to the process [64]. SUMO-mediated modification can be reversed by the action of deSUMOylating enzymes, which are also involved in the maturation of the SUMO precursor protein. SENPs belong to the most common family of protein deSUMOylases but, unrelated DESI1, DESI2, and USPL1 SUMO proteases exist as well [66]. Since SUMOylation is mostly restricted to the nucleus, it is not a surprising fact that SUMO is involved in many nuclear processes such as DNA damage response, genome integrity, transcription regulation, as well as preservation of protein stability and modulation of subcellular localization of the substrate proteins [67,68].

SUMOylation is a highly dynamic process enabling fast global changes in the SUMO status of the proteome in response to internal and external stimuli, often stress such as heat shock, nutrient depletion, genotoxic or oxidative stress [69,70,71,72]. This rapid adaptation is possible thanks to several mechanisms of regulation that can control SUMOylation levels. In addition to deSUMOylases, the SUMO-targeted ubiquitin ligase (STUbl) enzymes can modify global SUMOylation levels by binding to SUMO chains on proteins and poly-ubiquitinating them, eventually leading to their proteasome-mediated degradation. Moreover, a crosstalk between SUMOylation and other PTMs, such as ubiquitination or phosphorylation, has also been reported to affect the SUMOylation status [73,74]. The localization of the SUMO enzymatic machinery constitutes an additional critical factor for the modulation of the SUMOylation levels [64].

Hence, controlled SUMOylation is required for normal cell behavior. According to proteomics studies, between 1000 and 3000 human proteins are modified by SUMO. The identified SUMOylated proteins are implicated in almost all cellular processes [66]. A deregulation in SUMOylation dynamics has been associated with fibrotic disorders occurring in the heart, lung, and kidney, amongst other diseases [75,76,77]. And there is increasing evidence that SUMOylation might play a regulatory role in liver fibrosis too [78,79,80].

A recent study referred to Ubc9, the only existing SUMO E2 conjugating enzyme, as a potential therapeutic target for the prevention and treatment of liver fibrosis. Protein and mRNA expression levels of Ubc9 were described to be significantly upregulated in the LX-2 liver fibrosis in vitro model, and in the HepG2 and SMMC-7721 HCC cell lines. Interestingly, shRNA-mediated silencing of Ubc9 expression in activated LX-2 cells resulted in a decreased expression of α-SMA and type I collagen fibrosis markers, as well as a diminished secretion of IL-6 and TNF profibrotic cytokines. Additionally, downregulation of Ubc9 blocked cell cycle progression and promoted activated LX-2 cell cycle arrest in G_2_ phase. Importantly, an induction of apoptosis in activated LX-2 cells was detected after Ubc9 expression knockdown, mainly attributed to the abrogation of the canonical NF-κB signaling pathway, which is also a known target of SUMOylation [78].

Another piece of work placed the deSUMOylating enzyme SENP2 as a critical protein to attenuate CCl_4_-induced liver fibrosis in mice by inducing activated HSC apoptosis via suppression of Wnt/β-catenin signaling program. SENP2 protein and mRNA expression levels were found to be decreased both in vitro and in vivo in activated hepatic stellate cells (HSCs) during the CCl_4_-induced liver fibrosis mouse model, being those levels restored after removal of the damage stimulus. On the one hand, in vitro SENP2 overexpression resulted in a decreased α-SMA and COL1A1 protein expression in a TGF-β-activated hepatic stellate cell line. Moreover, increased expression of SENP2 reduced cell viability, favored cell cycle arrest in G_0_/G_1_ phase and induced apoptosis of the in vitro TGF-β-activated HSCs. On the other hand, siRNA-mediated silencing of SENP2 in TGF-β-activated HSCs induced α-SMA and COL1A1 protein expression, stimulated cell proliferation, and reduced apoptosis. Finally, the expression of the Wnt/β-catenin pathway members was downregulated upon SENP2 overexpression in TGF-β-activated HSCs, thus suggesting a therapeutic role of SENP2 in liver fibrosis [79].

Although it has not been specifically studied in the context of liver, various members of the TGF-β/Smad canonical pathway, which is common to fibrotic processes, have been found to be SUMOylated [66]. TGF-β type I receptor (TRβI/ALK5), whose phosphorylation and activation are mediated by TGF-β, is SUMOylated further enhancing the activation and modulation of the downstream Smad signaling cascade [73]. Furthermore, TGF-β signal transducers Smad proteins are also postranslationally modified by SUMOylation. For example, Smad4 SUMOylation protects it from its ubiquitination and subsequent proteasomal degradation [81]. Interestingly, Smad nuclear interacting protein 1 (SNIP1), a transcription repressor for both TGF-β and NF-κB signaling pathways, is a SUMO substrate. SNIP1 inhibits the TGF-β signaling by hampering the recruitment of p300 coactivator to the Smad complex, whereas SNIP1 SUMOylation attenuates its inhibitory effect on the TGF-β response further facilitating the expression of PAI-1 and MMP2 [82]. In summary, it is suggested that interfering in the SUMOylation of these proteins could be a potential strategy for the treatment of diseases induced by aberrant TGF-β signaling, which not only includes liver fibrosis but also HCC. Nevertheless, more focused research is needed regarding the impact of the TGF-β/Smad pathway SUMOylation in the particular context of liver fibrosis.

Conversely, a study highlights the importance of SUMOylation for liver fibrosis regression. Reduced glutathione (GSH) is implicated in many cellular processes including fibrogenesis. GSH protects against oxidative stress, which activates HSCs. Thus, high levels of GSH would maintain HSC in a quiescent state, and this requires SUMOylation of Nrf2 and MafG, which facilitate heterodimerization and activation of the antioxidant response element (ARE) located in the promoter region of many genes involved in the antioxidant defense, such as the GSH synthetic enzymes [80].

Finally, it has also been demonstrated that SUMO 1 and SUMO2/3 could play a role as autoantigens during PBC, since autoantibodies to these proteins have been detected in the sera of patients suffering from this autoimmune disease. Nonetheless, further research is needed in order to understand how the development of SUMO autoantibodies can lead to autoimmunity in PBC [83].

Overall, SUMOylation is a highly dynamic process which can have both beneficial and pathological consequences in the cellular physiology depending on the protein substrate, cell type, or context. Therefore, inhibition of global SUMOylation might not always be an ideal therapeutic strategy due to potential unforeseeable secondary effects. Alternatively, a more realistic rationale would involve the discovery and development of small molecules or peptidomimetics that block the protein–protein interactions between specific E3 SUMO ligases or SENPs and their substrates that are known to be altered in a diseased state.

### 2.3. Therapeutic Strategies Targeting Ubls Modifications in Liver Fibrosis

As a result of several studies in the last decades about the role of PTMs, specifically Ubl-mediated protein modifications, and their implication in disease, many therapeutic agents targeting these modifications have been developed lately (see Reviews [84,85,86,87]). Nevertheless, only a small fraction of these agents was tested in liver fibrosis.

Regarding ubiquitination, the role of the pharmacological inhibitor LDN 57444, an inhibitor of the deubiquitinase ubiquitin C-terminal hydrolase1 (UCHL1), was also evaluated and shown to block the progression of established fibrosis in the carbon tetrachloride (CCl_4_) injured mice [37]. In addition, Indole-3-carbinol (I3C), a naturally occurring compound generated from the hydrolysis of glucobrassicin and found in high concentrations in *Brassica* vegetables, was shown to induce apoptosis of HSC through RIP1 K63 de-ubiquitination by upregulating deubiquitinase CYLD [88].

Therapeutic strategies targeting NEDDylation in liver fibrosis have also been evaluated. As it was previously mentioned, pre-clinical studies in mouse models have shown that the small pharmacological inhibitor of NEDDylation, Pevonedistat, or MLN4924 [89], is able to revert liver fibrosis [61]. Pevonedistat (MLN4924) is a potent and selective NAE1 inhibitor that is currently undergoing several clinical trials to treat some leukemias and some types of solid organ cancer pathologies. Taking this into account, translation of Pevonedistat from pre-clinical mouse models to clinical trials for liver fibrosis treatment should be a fast process. Finally, to our knowledge, the role of the inhibition of the SUMOylation pathway or specific enzymes of this pathway in liver fibrosis has not been assessed to date.

## 3. Concluding Remarks

**Figure 1:**
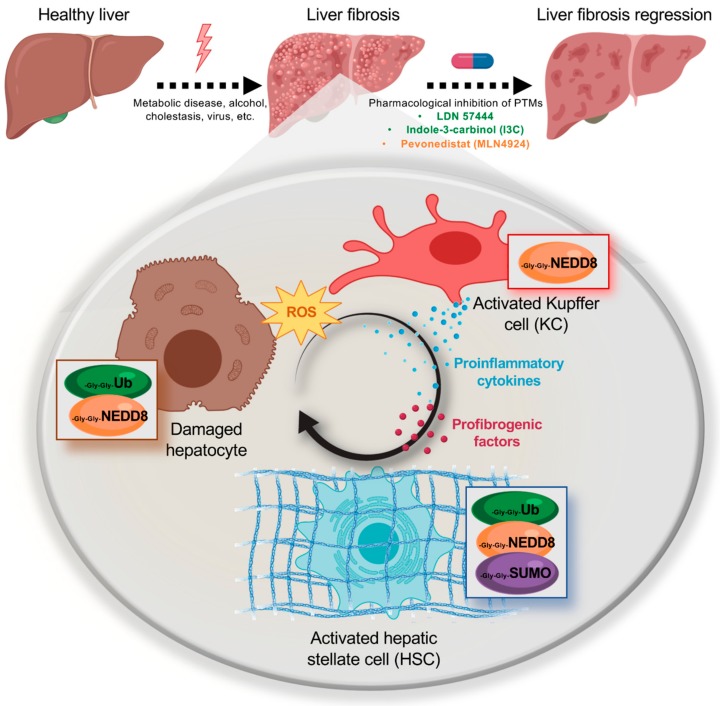
Schematic representation of the post-translational modifications (PTMs) described to date occurring in the main hepatic cell types involved during liver fibrosis, hepatocytes, Kupffer cells (KCs), and hepatic stellate cells (HSCs). Damage causing the transition from a normal healthy liver to a fibrotic liver are also referred, as well as the small-molecule inhibitors of PTMs that have resulted effective in the reversion of liver fibrosis.

In the last years, a big effort has been made on the study of the role of PTMs mediated by Ubl in liver fibrosis (Figure 1). In spite of the improved knowledge obtained on this highly dynamic and pan-cellular process of liver fibrosis and its regulation by Ubl PTMs, it is clear that novel tools need to be developed. As an example, in the last years, both tandem ubiquitin-binding entities (TUBEs) and SUMO-binding entities (SUBEs), were developed [90,91]. Briefly, TUBEs and SUBEs are recombinant proteins that comprise tandem repeats of either ubiquitin-associated (UBA) domains or SUMO-interacting motifs (SIMs) thereby recognizing with high affinity ubiquitin and SUMO molecules on modified proteins, respectively. In the liver context, the use of SUBEs has been used very recently to demonstrate the relevance of Liver Kinase B1 (LKB1) SUMOylation during the progression to Hepatocellular Carcinoma (HCC) highlighting its potential for the assessment of ubiquitinated and SUMOylated proteins in liver fibrosis [92]. Other option is to combine the use of transgenic mice with tagged Ubl PTMs where fibrosis is experimentally induced followed by isolation of the different hepatic populations playing a role on the progression of liver fibrosis. For instance, transgenic mouse models, specially dedicated to the study of the ubiquitin-proteasome system have been developed. This is the case of the mouse strains transgenic for a green fluorescent protein (GFP) reporter carrying a constitutively active degradation signal [93]. Moreover, Mayor and colleagues have developed a transgenic mouse expressing biotinylated ubiquitin and demonstrated its use for the isolation of ubiquitinated proteins from the liver by taking advantage of the specificity and strength of the biotin-avidin interaction [94]. Even though similar approaches for other Ubl modifications, such as NEDD8 and SUMO, have been used in cultured cells [95], novel in vivo approaches should be investigated. Importantly, studies to analyze the intermediates on the multiple types of hepatic cells participating in liver fibrosis and not only on HSC, as occurs in the majority of the studies found in literature, should be performed. And the reason for that is that to cure fibrosis is important not only to promote the apoptosis and the reversal of the activation of HSCs, but also to take out the injury insult mainly acting on liver hepatocytes, that is in fact driving the liver fibrosis cascade. Finally, regarding potential therapeutic approaches targeting Ubl PTMs, compelling evidence indicates that whereas NEDDylation inhibition provides a global mechanism for reversing liver fibrosis, with respect to ubiquitination and SUMOylation, we believe that potential therapeutic approaches in liver fibrosis should be more specific aiming at specific ligases with targets playing an important role in the fibrosis pathogenic processes.

## Figures and Tables

**Table 1:** Characterization of the structure, homology with ubiquitin, size, amino acid, and conservation between species (See Supplementary Materials for species disclosure) of ubiquitin [43] and the ubiquitin-like (Ubl) proteins, neural precursor cell expressed developmentally downregulated protein 8 (NEDD8) [44] and small ubiquitin-related modifier (SUMO) [45,46].

Ubls	Structure	Identity with Ubiquitin	Size (kDa)	Amino Acid	Highly Conserved between Species
Ubiquitin	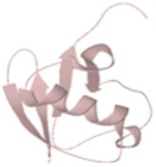	100	8.6	76	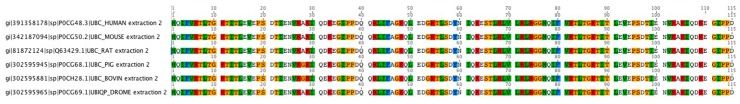
NEDD8	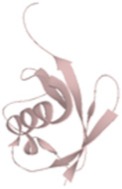	59	8	81	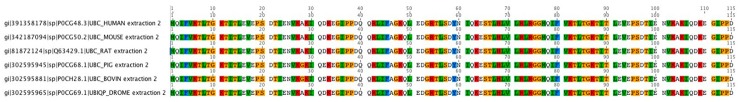
SUMO	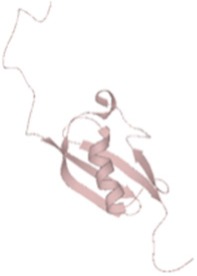 SUMO 1 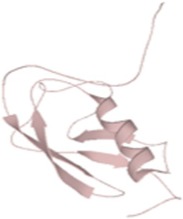 SUMO 2	18	≈12	≈100	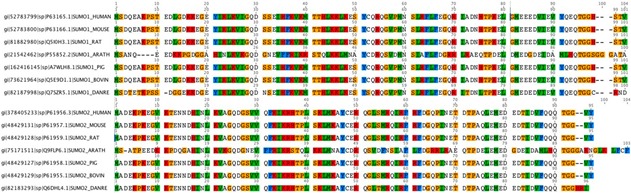

## Supplementary Materials

The following are available online at https://www.mdpi.com/2073-4409/8/12/1575/s1, Table S1: UNIPROT entry of the different species where homology of the Ubls was compared.
